# Prioritisation and risk ranking of epidemic-prone diseases for emergency preparedness and response in Eastern Africa using a multi-criteria decision analysis framework, 2023

**DOI:** 10.4102/jphia.v17i1.1496

**Published:** 2026-02-25

**Authors:** Neema Kamara, Motuma Guyassa, Georgios Theocharopoulos, Anouar S. Mohamed, Halifa M. Said, Despina Pampaka, Jonathan Suk, Lul P. Riek, Merawi Aragaw

**Affiliations:** 1 Africa Centres for Disease Control and Prevention, Addis Ababa, Ethiopia; 2 UK Health Security Agency, London, United Kingdom; 3 European Centre for Disease Prevention and Control, Solna, Sweden; 4 Africa Centres for Disease Control and Prevention, Nairobi, Kenya

**Keywords:** risk prioritisation, risk ranking, epidemic-prone diseases, emergency preparedness, emergency response, Eastern Africa, infectious disease, emerging infectious diseases

## Abstract

**Background:**

Resources to respond to emerging and re-emerging infectious disease emergencies are limited.

**Aim:**

To support preparedness planning and resource allocation, a prioritisation methodology was applied to rank epidemic-prone diseases in Eastern Africa.

**Setting:**

The study took place in the eastern region of Africa representing 14 member states.

**Methods:**

A multi-criteria decision analysis (MCDA) combined with a modified Delphi approach, adapted from the Africa Centres for Disease Control and Prevention (CDC’s) continental prioritisation framework was employed. A planning team of epidemiologists and infectious disease experts designed the exercise, implemented through a three-day workshop in Comoros from 9 May 2023 to 11 May 2023. The workshop convened 43 experts from Eastern Africa member states and partner organisations to rank diseases. Participants assessed each disease against 19 predefined criteria, grouped into four overarching domains: risk trajectory, epidemic potential, disease severity, and preparedness and medical countermeasures. Overall risk was calculated as the product of risk trajectory, epidemic potential, and disease severity.

**Results:**

Twenty-eight experts (88%, 28 of the eligible participants, *n* = 32) prioritised 22 potential epidemic-prone diseases. The top 10 were Ebola virus disease (risk = 12.7), Marburg virus disease (11.8), cholera (11.1), coronavirus disease 2019 (COVID-19) (9.9), influenza (8.9), measles (8.6), yellow fever (8.5), Crimean-Congo haemorrhagic fever (8.2), malaria (8.0) and Rift Valley fever (7.9). The unknown disease, defined as one caused by a hypothetical novel pathogen (often referred to as ‘Disease X’), Crimean-Congo haemorrhagic fever, Rift Valley fever and mpox received the lowest preparedness rating.

**Conclusion:**

Ebola virus disease (EVD), Marburg virus disease (MVD) and cholera ranked highest in risk, while unknown disease and zoonoses showed the lowest preparedness.

**Contribution:**

Amid current funding constraints, these findings provide evidence to guide Africa CDC and partners in strengthening emergency preparedness and response in Eastern Africa, highlighting priority diseases while underscoring the need for further analysis of capacity gaps.

## Introduction

The rising incidence of public health emergencies driven by natural disasters, as well as emerging and re-emerging infectious diseases, remains a significant concern in Africa.^1^ On average, the continent reports over 200 public health emergencies annually.^2^ A substantial proportion of these events are outbreaks of emerging infectious diseases, which vary in magnitude, geographical distribution and complexity. These outbreaks, along with other health emergencies, exert considerable societal and economic impacts, often necessitating extensive human, financial and material resources, thereby further straining already overburdened health systems.^3^

The inherent unpredictability of infectious disease surges, coupled with resource constraints and limited capabilities for timely risk prediction, underscores the urgent need for a planned, targeted and evidence-based approach to public health emergency management. Implementing such an approach is essential for reducing response delays and mitigating the morbidity and mortality associated with outbreaks. The International Health Regulations (IHR) emphasise risk assessment and prioritisation as core components of an effective Public Health Emergency Preparedness and Response (PHEPR) framework.^4^

Risk prioritisation and ranking have long been recognised as essential for identifying diseases that warrant heightened attention and dedicated resources to strengthen preparedness and response strategies.^5^ This process involves systematically identifying hazards and assessing and categorising disease-related risks based on their potential impact, likelihood of occurrence and transmission capacity. It underpins strategic preparedness planning, facilitates informed resource allocation and supports the effective implementation of targeted interventions aimed at mitigating the adverse consequences of outbreaks and pandemics.^5,6,7^ Furthermore, risk prioritisation provides a robust foundation for evidence-based decision-making, promotes dynamic and adaptive planning and ensures transparency and accountability in public health emergency management.

Given the dynamic nature of infectious diseases in Africa, continuous risk and disease prioritisation are imperative for the effective implementation of evidence-based preparedness strategies and response plans.

The Africa Centres for Disease Control and Prevention (Africa CDC) serves as the public health agency of the African Union, dedicated to strengthening the capacities of public health institutions and systems across the 55 African Union Member States.^8^ Its mission is to support the rapid detection and effective response to disease outbreaks and other health threats through an integrated, continent-wide framework encompassing surveillance, laboratory networks, disease prevention and control, emergency preparedness and response and public health research.

**FIGURE 1:**
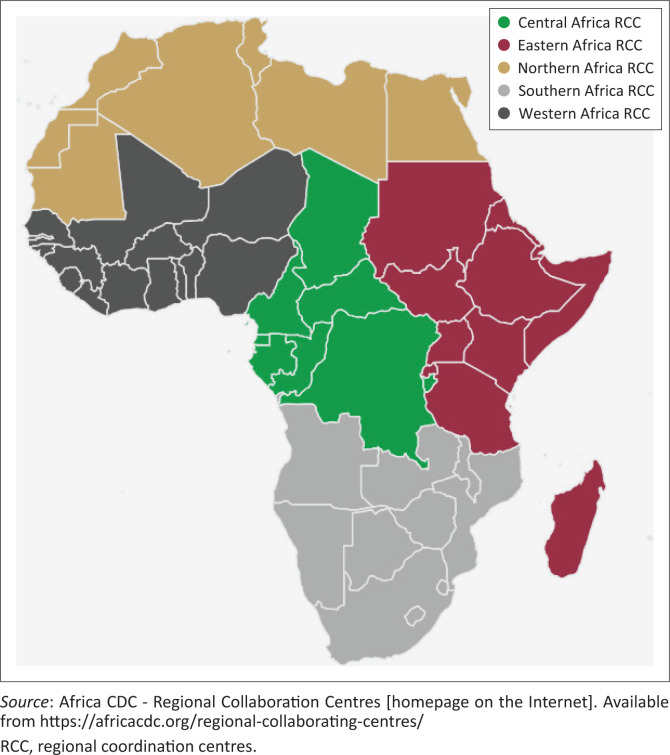
Africa Centres for Disease Control and Prevention regional coordination centres, 2025.

To enhance its operational efficiency and ensure timely support to Member States, Africa CDC established five Regional Coordination Centres (RCCs) in Eastern, Southern, Western, Central and Northern Africa (Figure 1),^9^ serving as decentralised hubs for implementing programmes and enabling regionally tailored responses to public health threats.

Building on the Africa CDC’s first continental-level prioritisation of epidemic-prone diseases in 2022,^10^ this process was adapted regionally to reflect diverse epidemiological profiles and public health challenges. Eastern Africa faces recurrent infectious disease outbreaks, impacts of natural disasters and climate change, zoonotic threats and a high burden of foodborne illnesses, compounded by limited resources, suboptimal surveillance, limited cross-border collaboration, fragmented data and inadequate public health infrastructure. In this context, a regional disease prioritisation exercise was carried out in 2023 to guide strategic planning, strengthen preparedness and enhance the capacity of Eastern Africa to respond effectively to emerging and epidemic-prone threats.

This paper presents the methodological approach employed for prioritising and ranking epidemic-prone diseases in Eastern Africa and summarises the key outcomes of the exercise.

## Research methods and design

The regional risk prioritisation process was adapted from the Africa CDC’s continental framework, which is based on the European Centre for Disease Prevention and Control (ECDC) risk ranking methodology utilising multi-criteria decision analysis (MCDA).^10,11^ Multi-criteria decision analysis provides a structured, transparent and reproducible approach for evaluating and comparing options using multiple decision-making criteria.^12^ To enhance the rigour of the process and ensure expert consensus, the modified Delphi technique was applied to systematically collect, review and harmonise expert input on disease selection, ranking criteria and prioritisation outcomes. The modified Delphi process is a systematic and structured method designed to facilitate consensus among a group of experts on a specific topic.^13^

**FIGURE 2:**
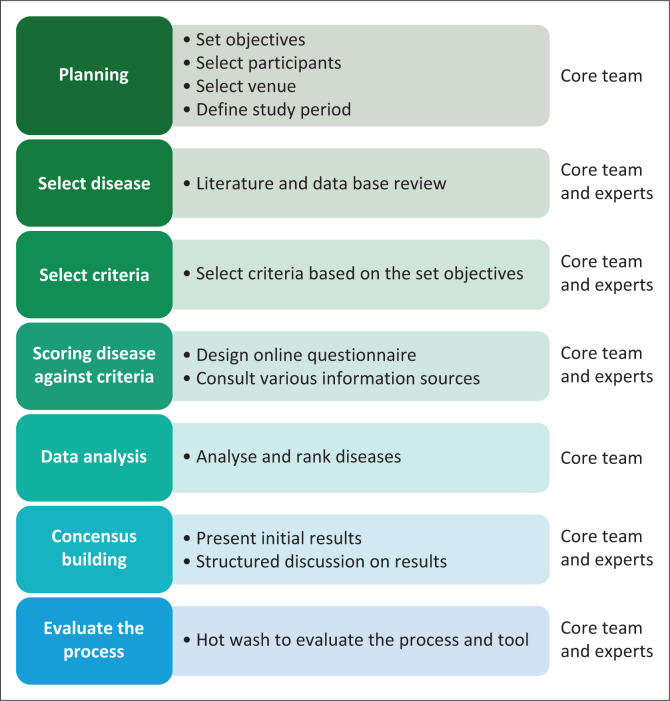
Risk ranking process, Africa Centres for Disease Prevention and Control, May 2023.

The Africa CDC risk ranking methodology involves seven key steps: (1) Planning; (2) disease selection; (3) definition of ranking criteria; (4) scoring of diseases against the criteria; (5) disease ranking; (6) consensus building; and (7) evaluation of the process (Figure 2).

### Planning

A team of five epidemiologists and infectious disease experts from Africa CDC and ECDC designed the exercise, tailoring the continental methodology to Eastern Africa’s context. A 3-day in-person workshop was held in Comoros from 09 May 2023 to 11 May 2023 to orient participants to the objectives, methodology, conceptual framework and tool used. Comoros was chosen as one of the countries in Eastern Africa for regional representation rather than on the basis of scientific criteria. The selection of the venue did not influence or affect the study outcomes. The workshop enhanced transparency, encouraged collaboration and fostered consensus-building among regional stakeholders.

### Workshop participants

A total of 43 experts participated, representing Eastern Africa Member States, regional bodies and global partners: Comoros (*n* = 6), Madagascar (*n* = 2), Seychelles (*n* = 2), Mauritius(*n* = 2), Djibouti (*n* = 2), Ethiopia (*n* = 2), Somalia (*n* = 2), Kenya (*n* = 2), Tanzania (*n* = 2), Uganda (*n* = 2), Rwanda (*n* = 1), Africa CDC HQ (*n* = 5), Eastern RCC (*n* = 4), Central RCC(*n* = 1), ECDC (*n* = 2), Comoros World Health Organization (WHO) country office (*n* = 2), United Nations Children’s Fund (UNICEF) (*n* = 2), African Society for Laboratory Medicine (ASLM) (*n* = 1) and the East, Central and Southern Africa Health Community (ECSA-HC) (*n* = 1). Participants included epidemiologists (*n* = 17), laboratory scientists (*n* = 7), infectious disease experts (*n* = 6), zoonosis experts (*n* = 8) and vaccine and supply chain experts (*n* = 5). To ensure the selection of appropriate participants, the Ministries of Health were requested to nominate subject matter experts, prioritising individuals with demonstrated technical expertise, epidemiological proficiency and contextual knowledge of national and regional public health risks.

### Selection of diseases

Six infectious disease outbreak data sources, including the Africa CDC Event-Based Management System (EMS) and Member States supported by the Eastern RCC, were triangulated to identify outbreaks reported in Eastern Africa from 2019 to 2022. An outbreak was defined as the occurrence of disease cases above what would be normally expected in a defined community, geographical area or season.^14^ Countries were requested via email to provide details for each outbreak, including the number of cases and deaths, locations, frequency and duration, and the causative agent. After reviewing all sources, a total of 28 disease- or pathogen-specific outbreaks were identified during the specified period.

During a 3-day workshop, this initial list of 28 diseases was presented, and a structured discussion was facilitated to reach consensus on the final selection of diseases for prioritisation. Based on predefined criteria, the stated experts selected 22 diseases for ranking (Table 1). The selection was guided by the diseases’ potential public health impact assessed through multiple dimensions: frequency of occurrence, severity as indicated by case fatality ratios, extent of geographical spread (including cross-border transmission), overall disease burden (incidence and mortality) and the scale of response required.

**TABLE 1:** Selection of priority diseases for prioritisation in the Eastern Africa risk ranking, May 2023.

Acute haemorrhagic fevers	Vaccines preventable diseases	Water-borne	Zoonoses	Vector-borne	Respiratory infections	Others
Ebola virus disease (EVD)	Measles	Cholera	Mpox	Malaria	Influenza caused by new subtype	Unknown disease
Crimean-Congo haemorrhagic fever (CCHF)	Meningococcal disease (*Neisseria meningitidis*)	-	Rabies	Yellow	COVID-19	-
Marburg virus disease (MVD)	Wild poliomyelitis	-	Anthrax	Chikungunya	-	-
	Vaccine-derived poliomyelitis	-	Leptospirosis	Dengue fever	-	-
	-	-	Rift Valley fever (RVF)	-	-	-
	-	-	Brucellosis	-	-	-
	-	-	Plague	-	-	-

COVID-19, coronavirus disease.

### Criteria

The same 19 criteria developed in 2022 for continental risk prioritisation were applied, grouped into four overarching categories: risk trajectory, epidemic potential, disease severity, preparedness and countermeasures (Table 2). These criteria had been reviewed and validated during two multisectoral consultative workshops with key stakeholders in May 2022.^10^

**TABLE 2:** Risk ranking criteria, Eastern Africa, 2023.

Category	Criterion	Description
Risk trajectory	Probability of the pathogen circulating among humans in the African region in the next 5 years	Probability that a pathogen enters Africa in the next 5 years, either through import of products, animals or humans carrying the pathogen or vectors that harbour the pathogen. When a pathogen is already present or likely to be introduced, this is represented by the ‘high’ level. This criterion excludes consideration of pathogens in laboratories.
	Probability that the threat associated with this pathogen increases in the next 5 years in Africa	Worsening of the threat can occur through various mechanisms, including the evolution of new pathogen traits (e.g., virulence, enhanced transmissibility in humans, antimicrobial resistance), changing vector habitats (e.g. because of climate change), changes in animal reservoirs or changes in human mobility.
Epidemic potential	Transmissibility of the pathogen in comparison to other pathogens being prioritised	This criterion assesses how easily a pathogen could spread in Africa, assuming that the pathogens are already present. Transmissibility is determined by many factors, including mode of transmission, the infectivity of the pathogen, the contagiousness of infected and exposed individuals and social mixing patterns. The basic reproduction number, R_*o*_, is the metric used to assess transmissibility.
	Population susceptibility: how many regions in Africa have high pools of susceptible populations in comparison to the other pathogens being prioritised	This criterion attempts to estimate the population susceptibility to severe disease to a given pathogen, through considering how many regions are likely to have high pools of pockets of susceptible populations.
	Probability of the pathogen causing a cross-country outbreak: what is the likelihood, compared to other pathogens being prioritised, that the pathogen could lead to a cross-country outbreak	This criterion assesses the likelihood of an outbreak originating in one country leading to an outbreak in a neighbouring country, thereby increasing the complexity and challenge of the response.
Disease severity	Peak infection fatality rates	Proportion of cases that are fatal from the disease under consideration during the peak of a possible epidemic. It depends on the pathogen causing the disease under consideration, the health state of the patients and available medical countermeasures.
	Proportion of cases that lead to severe disease	The impacts of non-fatal severe disease outcomes can be substantial. Severe disease implies severe symptoms that could require hospitalisation and/or lead to longer-term sequalae.
	Estimated economic impact of an outbreak of 1000 cases of the disease	Total impact in monetary terms of a hypothetical outbreak of 1000 cases from this pathogen, considering overall societal costs (i.e., costs for the society as a whole). These costs include direct cost to the healthcare system and to preparedness and response; and indirect costs related to productivity losses, tourism losses and trade losses. The costs are expressed as total estimated costs per 1000 cases per year, reflecting the distribution of health outcomes and associated costs per 1000 cases of a given infection.
	Estimated social impact of an outbreak of the disease in comparison to other pathogens being prioritised	Outbreaks of certain diseases may have significant social impacts because of societal fear, stigmatisation, the need to adhere to public health and social measures, or other factors. This criterion should be assessed purely by comparing the relative possibilities for negative social impacts across the different pathogens being prioritised.
Preparedness	Level of public health preparedness to deal with an outbreak of the pathogen in comparison to other pathogens being prioritised	Public health preparedness for specific pathogens depends upon capacities for detecting, identifying and responding to outbreaks caused by this pathogen. In addition, previous experience dealing with outbreaks of a given pathogen, the existence of preparedness plans for this pathogen or prior simulation exercises or after-action reviews focused on this pathogen may be indicative of greater capabilities for handling an outbreak of this pathogen.
Medical countermeasures (Vaccines)	Availability of vaccines	This criterion assesses the extent to which vaccines are available to be administered to target populations during a public health response. If no vaccines for a pathogen exist, or if these are not available in the jurisdiction of an outbreak, then ‘very low’ shall be selected.
	Effectiveness of vaccines	This criterion assesses the effectiveness of the vaccine against severe disease and mortality. While the effectiveness against onwards transmission, sterilising immunity, is also highly desirable, this is not explicitly considered in this criterion.
	Anticipated societal acceptance of vaccines	Ultimately, vaccines will only be effective if a sufficient proportion of the population accepts to receive them. This criterion assesses the extent to which target populations for vaccination would accept vaccination.
Medical countermeasures (Pharmaceutical)	Availability of pharmaceutical countermeasures	This criterion assesses the extent to which pharmaceutical interventions (excluding vaccines) relevant to the pathogen are available to be administered to target populations during a public health response. If no pharmaceutical interventions for a pathogen exist, or if these are not available in the jurisdiction of an outbreak, then ‘very low’ shall be selected.
	Effectiveness of pharmaceutical countermeasures	In this criterion, the effectiveness of available medical interventions to reduce severe outcomes including fatality is assessed. If the answer to 13a was ‘no measures available’, then this field should not be filled in.
	Anticipated societal acceptance of and adherence to pharmaceutical countermeasures	This criterion assesses the estimated proportion of ill patients that would accept medicines to treat their disease.
Non-medical countermeasures (public health and social measures [non-pharmaceutical])	Availability of non-pharmaceutical measures for controlling an outbreak (e.g., case isolation, contact tracing, mosquito nets, etc.)	This criterion assesses the extent to which non-pharmaceutical interventions relevant to the pathogen (mosquito nets, physical distancing, facemasks, etc.) can be administered to target populations during a public health response. If no non-pharmaceutical interventions for a pathogen exist, or if these are not available in the jurisdiction of an outbreak, then ‘very low’ shall be selected.
	Effectiveness of non-pharmaceutical measures for controlling an outbreak	In this criterion, the effectiveness of available and relevant non-pharmaceutical measures in reducing transmission and exposure to the pathogen is assessed.
	Anticipated social acceptance ofnon-pharmaceutical measures	Ultimately, non-pharmaceutical interventions (NPIs) will only be effective if a sufficient proportion of the population adheres to them. This criterion assesses the extent to which target populations might be expected to adhere to recommended NPIs.

### Scoring the diseases

Eligible participants (*n* = 32), defined as individuals with relevant expertise and contextual knowledge, excluding workshop facilitators and meeting administrator, independently scored the 22 diseases against the 19 predefined criteria. Each criterion was scored using a standard four-point scale (1 = very low, 4 = high) to ensure consistency and comparability of responses (Table 3).^11^ Data were collected using the online Kobotoolbox questionnaire. Experts were encouraged to consult scientific literature and online resources to ensure evidence-based scoring.

**TABLE 3:** Scoring levels of criteria, Eastern Africa risk ranking, May 2023.

Criterion	Range	Value
Probability of the pathogen circulating among humans in the African region in the next 5 years	< 1%	1
	1% – 10%	2
	10% – 90%	3
	> 90%	4
Probability that the threat associated with this pathogen increases in the next 5 years in Africa	< 1%	1
	1% – 10%	2
	10% – 90%	3
	> 90%	4
Transmissibility of the pathogen in comparison to other pathogens being prioritised	R_*o*_ < 1	1
	R_*o*_ between 1 and 2	2
	R_*o*_ between 2 and 4	3
	R_*o*_ > 4	4
Population susceptibility: how many regions in Africa have high pools of susceptible populations in comparison to the other pathogens being prioritised	< 1%	1
	1% – 10%	2
	10% – 90%	3
	> 90%	4
Probability of the pathogen causing a cross-country outbreak: what is the likelihood, compared to other pathogens being prioritised, that the pathogen could lead to a cross-country outbreak	< 1%	1
	1% – 10%	2
	10% – 90%	3
	> 90%	4
Peak infection fatality rates	< 0.1%	1
	0.1% – 1.0%	2
	1% – 10%	3
	> 10%	4
Proportion of cases that lead to severe disease	< 1%	1
	1% – 5%	2
	5% – 50%	3
	> 50%	4
Estimated economic impact of an outbreak of 1000 cases of the disease	< 1 million USD	1
	1–10 million USD	2
	10–100 million USD	3
	> 100 million USD	4
Estimated social impact of an outbreak of the disease in comparison to other pathogens being prioritised	Very low	1
	Low	2
	Medium	3
	High	4
Level of public health preparedness to deal with an outbreak of the pathogen in comparison to other pathogens being prioritised	No plan exists	1
	Plan in development	2
	Plan without simulation exercises	3
	Plan tested and revised through simulation exercise	4
Availability of vaccines	No vaccines available	1
	Sufficient vaccine supply to reach < 40% of target population coverage	2
	Sufficient vaccine supply to reach 40% – 70% of target population coverage	3
	> 70% target population coverage	4
Effectiveness of vaccines	< 40%	1
	40% – 70%	2
	70% – 90%	3
	> 90%	4
Anticipated societal acceptance of vaccines	< 25%	1
	25% – 50%	2
	50% – 75%	3
	> 75%	4
Availability of pharmaceutical countermeasures	No measures available	1
	Sufficient supply to reach < 40% of target population coverage	2
	Sufficient supply to reach 40% – 70% of target population coverage	3
	> 70% target population coverage	4
Effectiveness of pharmaceutical countermeasures	< 50%	1
	50% – 70%	2
	70% – 90%	3
	> 90%	4
Anticipated societal acceptance of and adherence to pharmaceutical countermeasures	< 40%	1
	40% – 70%	2
	70% – 90%	3
	> 90%	4
Availability ofnon-pharmaceutical measures for controlling an outbreak(e.g., case isolation, contact tracing, mosquito-nets, etc.)	< 50% target population coverage	1
	50% – 70% target population coverage	2
	70% – 90% target population coverage	3
	> 90% target population coverage	4
Effectiveness ofnon-pharmaceutical measures for controlling an outbreak	< 50%	1
	50% – 70%	2
	70% – 90%	3
	> 90%	4
Anticipated social acceptance of non-pharmaceutical measures	< 25%	1
	25% – 50%	2
	50% – 75%	3
	> 75%	4

USD, Unites States dollar.

### Analysis and ranking of diseases

The arithmetic of the score was calculated using equal weights for all criteria. Scores for risk trajectory and epidemic potential were combined into a composite measure called epidemic potential. Descriptive analysis, including means and interquartile ranges (25th–75th percentiles) of individual expert scores, was performed for epidemic potential, disease severity and preparedness and countermeasures. Expert agreement was assessed using box plots, with narrower interquartile ranges indicating higher consensus. The risk score was calculated by multiplying the mean epidemic potential by the mean disease severity, expressed as risk = epidemic potential × disease severity. Diseases were subsequently ranked from highest to lowest risk, and rankings were compared against levels of preparedness. Sensitivity analysis was performed and showed no statistically significant differences in the outcomes across the group (*p* > 0.05).

### Presentation of outcomes and consensus building

On the workshop’s final day, experts reviewed the results to reach consensus on the final prioritisation, ensuring that the top-ranked diseases represent the most urgent public health threats requiring targeted interventions and resource allocation.

### Process evaluation

A feedback survey was administered to assess the relevance, usability, adaptability and effectiveness of the prioritisation tool in meeting its objectives. Of the 20 participants who completed the evaluation survey, 13 were Anglophone and seven were Francophone respondents. Nineteen participants (95%) agreed that the criteria were clear, the tool was easy to navigate, and the overall results were appropriate. Participants also noted that, although the findings accurately reflect regional-level priorities, they may not necessarily correspond to priorities at the country level.

### Ethical considerations

Approval to conduct the exercise was obtained from both Africa Centres for Disease Control and Prevention (Africa CDC) and European Centre for Disease Prevention and Control (ECDC). No ethical approval was needed as it was not perceived as a study. All participants contributed voluntarily and were informed about the purpose of the study. No identifiable personal information was collected, and all responses were anonymised to ensure confidentiality.

## Results

A total of 28 experts completed the online questionnaire. Their responses revealed marked variation in the perceived mean scores for epidemic potential, disease severity and preparedness across 22 priority infectious diseases. Cholera, coronavirus disease 2019 (COVID-19) and Ebola virus disease (EVD) were identified as having the highest epidemic potential (mean scores > 3.2), and EVD (3.8), Marburg virus disease (MVD) (3.7) and cholera (3.2) were rated as the most severe. The lowest preparedness levels were reported for unknown disease (mean score 1.6), mpox, Rift Valley fever (RVF) and Crimean-Congo haemorrhagic fever (CCHF) (each with a mean score of 2.0), highlighting critical gaps in readiness for these threats (Table 4).

**TABLE 4:** Distribution of average means for epidemic potential, disease severity, overall risk and preparedness level of selected priority diseases, Eastern Africa risk ranking, May 2023.

Diseases	Epidemic potential	Disease severity	Overall risk	Preparedness
EVD	3.3	3.8	12.7	2.3
MVD	3.2	3.7	11.8	2.1
Cholera	3.5	3.2	11.1	2.7
COVID-19	3.3	3.0	9.9	2.9
Influenza	3.2	2.8	8.9	2.2
Measles	3.3	2.6	8.6	3.0
Yellow fever	3.0	2.8	8.5	2.5
CCHF	2.6	3.1	8.2	2.0
Malaria	3.1	2.6	8.0	2.7
RVF	2.9	2.8	7.9	2.0
Dengue fever	3.0	2.5	7.4	2.2
Unknown disease	2.6	2.8	7.4	1.6
Meningococcal disease	2.7	2.7	7.1	2.5
Rabies	2.3	2.9	6.8	2.2
Wild poliomyelitis	2.5	2.5	6.4	2.7
Mpox	2.5	2.5	6.3	2.0
Vaccine-derived poliomyelitis	2.6	2.4	6.3	2.7
Chikungunya	2.9	2.2	6.2	2.1
Plague	2.1	2.8	5.8	2.1
Anthrax	2.3	2.4	5.6	2.1
Brucellosis	2.3	2.0	4.7	2.1
Leptospirosis	2.1	2.1	4.4	2.1

EVD, Ebola virus disease; MVD, Marburg virus disease; CCHF, Crimean-Congo haemorrhagic fever; RVF, Rift Valley fever; COVID-19, coronavirus disease.

The top 10 highest risk-ranked diseases were EVD (risk = 12.7), MVD (11.8), cholera (11.1), COVID-19 (9.9), influenza (8.9), measles (8.6), yellow fever (8.5), CCHF (8.2), malaria (8.0) and RVF (7.9) (Table 4).

**FIGURE 3:**
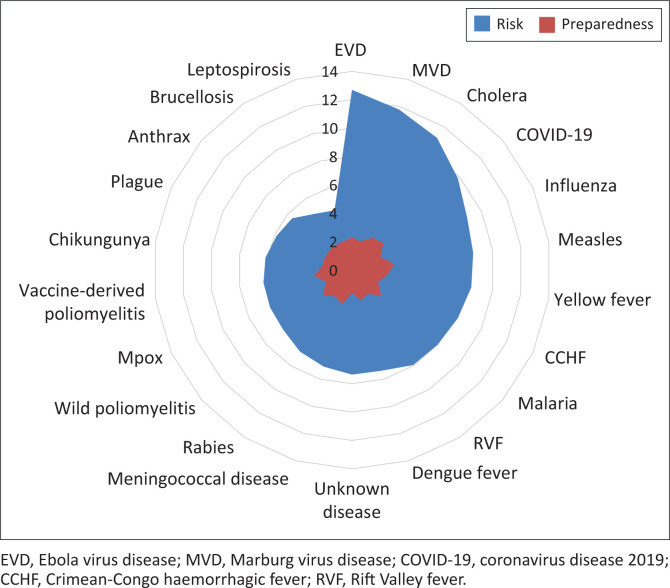
Risk scores and preparedness levels of selected priority diseases, Eastern Africa risk ranking, May 2023.

The results also showed a clear mismatch between risk and preparedness levels for several high-priority diseases, underscoring the need for targeted investment in medical and non-medical countermeasures, especially for emerging and re-emerging threats. EVD (risk = 12.7), MVD (11.8) and cholera (11.1) were ranked as the highest-risk diseases, yet showed only moderate preparedness levels (2.3, 2.1 and 2.7 respectively). Several emerging threats, including CCHF (risk = 8.2; preparedness mean = 2.0), RVF (risk = 7.9; preparedness mean = 2.0) and mpox (risk = 6.3; preparedness mean = 2.0), had relatively moderate risk scores but low preparedness, indicating major response gaps. Measles (risk = 8.6; preparedness mean = 3.0) and COVID-19 (risk = 9.9; preparedness mean = 2.9) showed better alignment between risk and preparedness, likely reflecting ongoing control efforts (Figure 3).

**FIGURE 4:**
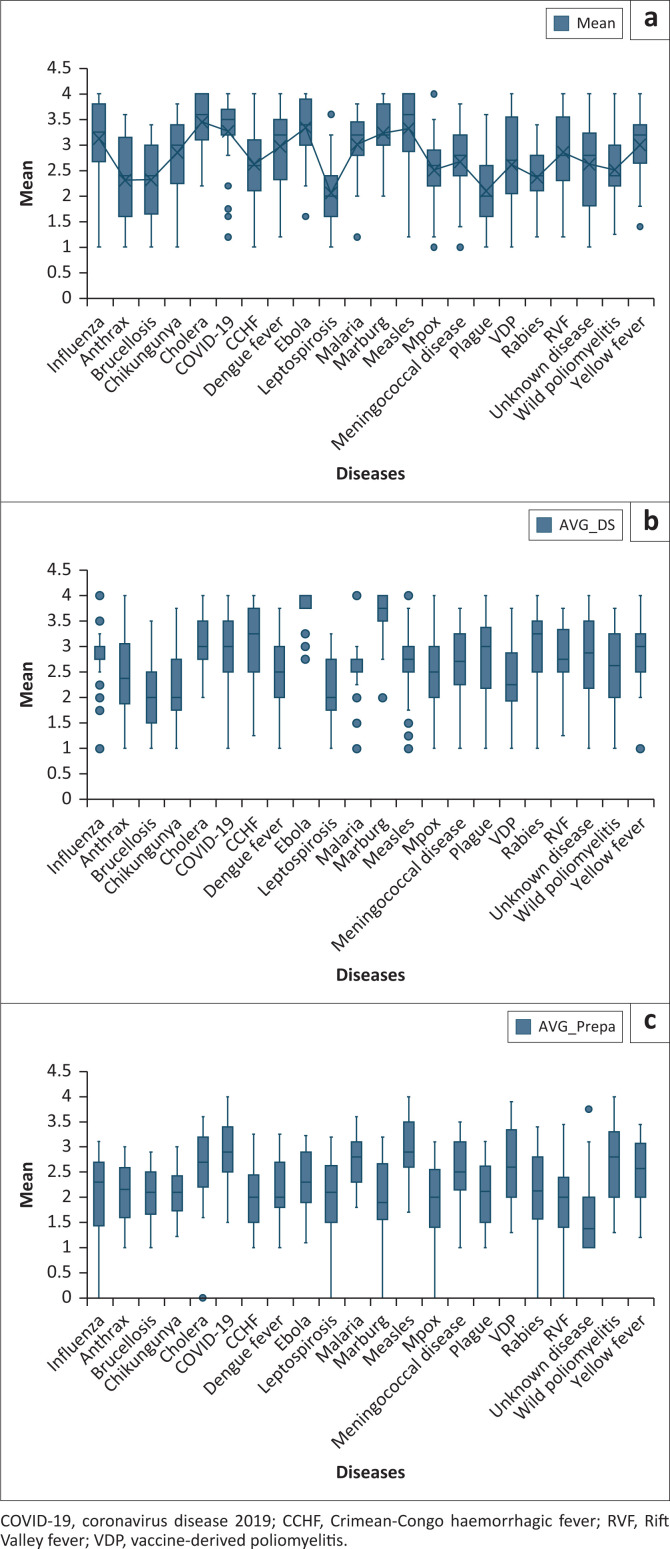
Level of agreement among experts on scores of priority diseases, Eastern Africa risk ranking, May 2023. (a) Epidemic potential. (b) Disease severity. (c) Preparedness and countermeasures.

The boxplots illustrate variability in expert assessments of epidemic potential, disease severity and preparedness levels across 22 priority diseases. For epidemic potential, diseases such as COVID-19, malaria, mpox and rabies exhibited narrow interquartile ranges, indicating strong expert agreement. In contrast, anthrax, brucellosis, vaccine-derived poliomyelitis and unknown diseases showed wider distributions, suggesting lower consensus. The presence of multiple outliers for COVID-19 may reflect regional differences in experience or ongoing uncertainty regarding its future trajectory. Regarding disease severity, EVD, malaria, MVD and influenza demonstrated narrow interquartile ranges, reflecting consensus on their clinical impact. However, the frequent outliers for MVD and measles highlight divergent expert views, likely shaped by context-specific burden or prior experiences. Preparedness scores were broadly consistent across disease, clustering around moderate levels. Measles and COVID-19 received the highest ratings, while unknown diseases received the lowest, with the narrow interquartile range observed suggesting uniform expert concern regarding response capacity. These findings underscore the importance of accounting for both consensus and variability in expert assessments when informing preparedness and prioritisation strategies (Figure 4).

## Discussion

The top 10 ranked diseases in Eastern Africa included, in order: EVD, MVD, cholera, COVID-19, influenza, measles, yellow fever, CCHF, malaria and RVF. While most of these diseases are zoonotic in origin, others – such as cholera, measles and malaria – are not. Reports indicate that these diseases remain prevalent and continue to cause significant public health challenges in the region.^14,15,16,17,18^ This finding aligns with the 2017 World Health Organization’s recent list of viral and bacterial families with the highest potential to cause future epidemics and pandemics.^18^ The results also show similarities, with some distinctions, when compared to the continental-level risk prioritisation.^10^ Of note, in contrast to the continental prioritisation, measles, malaria and RVF ranked higher in the Eastern region’s prioritisation. These findings underscore the critical need to prioritise resources, workforce training and contingency planning tailored to the specific health threats and risks prevalent in Eastern Africa. The concurrent burden of emerging zoonotic diseases – such as EVD, MVD, CCHF and RVF – alongside persistent endemic diseases such as malaria, influenza and measles necessitates a dual-pronged approach. The findings further indicated that zoonotic diseases such as RVF, CCHF and mpox are associated with low levels of preparedness, highlighting systematic vulnerabilities. Therefore, strategies should integrate epidemic preparedness with the strengthening of routine disease control programmes. To improve regional response capacity, it is essential to ensure the prepositioning of critical supplies, including vaccines, therapeutics, diagnostics and personal protective equipment (PPE). Additionally, sustained investments in health infrastructure, workforce development and the establishment of clear policies and coordination frameworks are vital to enable timely and effective responses to these priority health threats. Strengthening collaborations and establishing research networks at both national and regional levels, as well as integrating a One Health approach, will be instrumental in enhancing preparedness and response capacities. Given the transboundary nature of many prioritised diseases, cross-border collaboration is particularly important. Coordinated surveillance, data sharing, joint outbreak investigations and harmonised response strategies among neighbouring countries are essential to contain outbreaks at their source and prevent wider regional transmission. Enhancing these mechanisms will contribute significantly to regional health security and build resilience against both current and future public health emergencies.

Despite substantial investments in vaccination and control efforts, experts continued to prioritise COVID-19, even though it was no longer classified as a global health emergency at the time of the workshop.^19^ This reflects ongoing concerns about its continued pandemic potential, given the virus’s capacity for rapid mutation, seasonal resurgence and antigenic variation – particularly in the context of limited vaccine access and the weakening of sustained control measures in many countries.^20,21,22^ Severe acute respiratory syndrome coronavirus 2 (SARS-CoV-2) virus still evolves in humans and undergoes genetic mutations as a result of immune pressure exerted by factors such as herd immunity, prior infections, therapeutic interventions, vaccination and exposure to neutralising antibodies. These mutations can enhance viral infectivity and transmissibility while also reducing the efficacy of neutralisation by monoclonal antibodies, convalescent plasma or vaccine-induced immunity.^23^ Furthermore, mutations may also lead to failure of detection by molecular diagnostic tests, leading to delayed diagnosis, increased community spread and delayed treatment.^23^

The interplay between risk and preparedness is critical in guiding effective public health responses. High-risk diseases – characterised by their epidemic potential and severity – demand proportionately greater preparedness efforts, including robust surveillance systems, rapid response capacity, readily available medical and non-medical countermeasures and resource allocation. However, preparedness levels are not always commensurate with actual or perceived risk. In some cases, diseases with high perceived risk may be under-resourced, while others benefit disproportionately from pre-existing systems or global attention, e.g., measles and polio. This mismatch underscores the need for dynamic, evidence-based prioritisation frameworks that integrate both evolving risk profiles and existing preparedness capacity. For instance, the risk posed by filoviruses such as EVD and MVD in East Africa is high, with substantial response implications. However, preparedness for such outbreaks remains suboptimal, as demonstrated by recent epidemics in Uganda, Rwanda and Tanzania.^24,25,26^ There is a need to improve access to prequalified vaccines and therapeutics and to strengthen research-embedded, One Health approaches to better understand the origins and drivers of outbreaks. Aligning preparedness with risk through such measures is essential to improving outbreak prevention, detection and response in the region. The outliers in disease severity scores reflect heterogeneity in expert perceptions, shaped by differences in professional background, field experience and contextual knowledge. This variation underscores the multi-dimensional nature of disease severity, encompassing clinical, economic and social impacts.

This exercise employed the MCDA methodology, which is well suited for prioritisation efforts involving multiple criteria. A range of criteria was selected to comprehensively reflect the objectives of the exercise, and the approach enabled multidisciplinary participation. The tool incorporated parameters to evaluate both risk and high-level emergency preparedness interventions. In settings where data may be limited, such risk-ranking tools are particularly valuable, allowing experts to apply their knowledge and judgement in prioritising public health threats. Extensive expert consultation contributed to stakeholder acceptance, endorsement and confidence in the results. This exercise complements existing risk-ranking frameworks, such as the WHO Strategic Tool for Assessment of Risks (STAR), by providing a more granular, disease-specific analysis using a multi-criteria decision approach. Its methodological innovation and contextual relevance make it both novel and fit for purpose in guiding evidence-based preparedness and response strategies.

Despite its usefulness, this exercise has some limitations. Firstly, the final risk ranking scores represent relative, not absolute, risk and are based on expert judgement rather than direct epidemiological measures. They reflect perceived threats informed by current knowledge, recent outbreak history and potential public health impact, rather than actual incidence rates. Secondly, assuming equal weights for all criteria may have reduced analytical precision. A weighted approach could yield more nuanced prioritisation. Thirdly, expert assessments may have been influenced by recent COVID-19 and EVD outbreaks in the region, introducing potential bias. Finally, the absence of real-time data and modelling constrained accurate risk estimation, increasing reliance on expert opinion. While the results reflect regional priorities, they may not fully capture country-specific needs. Even within a shared region, countries can vary significantly in disease epidemiology, health system capacity and contextual factors. This highlights the importance of conducting national-level disease prioritisation exercises. The knowledge and experience gained from this regional process can serve as a foundation for country-specific efforts, tailored to distinct objectives and contexts.

## Conclusion

The findings of this exercise highlight priority diseases for Africa CDC and partners, providing critical evidence to strengthen preparedness and response in Eastern Africa. Institutionalising this prioritisation process across all levels and updating it every 2–3 years will ensure strategies remain targeted, adaptive and effective. Further analysis of capacity and capability gaps related to priority diseases is essential to inform evidence-based, coordinated and sustainable preparedness strategies.
